# Comparing Genetic Risk and Clinical Risk Classification in Luminal-like Breast Cancer Patients Using a 23-Gene Classifier

**DOI:** 10.3390/cancers14246263

**Published:** 2022-12-19

**Authors:** Chi-Cheng Huang, Ting-Hao Chen, Liang-Chih Liu, Chiun-Sheng Huang, Ji-An Liang, Yu-Chen Hsu, Chia-Ming Hsieh, Sean-Lin Huang, Kuan-Hui Shih, Ling-Ming Tseng

**Affiliations:** 1 Comprehensive Breast Health Center, Department of Surgery, Taipei Veterans General Hospital, Taipei 11217, Taiwan; 2 Institute of Epidemiology and Preventive Medicine, College of Public Health, National Taiwan University, Taipei 106170, Taiwan; 3 Amwise Diagnostics Pte. Ltd., Singapore 069547, Singapore; 4 Department of General Surgery, China Medical University Hospital, Taichung 40454, Taiwan; 5 College of Medicine, China Medical University, Taichung 40402, Taiwan; 6 Department of Surgery, National Taiwan University Hospital, Taipei 300600, Taiwan; 7 Department of Radiation Oncology, China Medical University Hospital, Taichung 40454, Taiwan; 8 Department of General Surgery, Ditmanson Medical Foundation Chia-Yi Christian Hospital, Chia-Yi 60002, Taiwan; 9 Department of General Surgery, Taiwan Adventist Hospital, Taipei 105520, Taiwan; 10 Faculty of Medicine, College of Medicine, National Yang-Ming University, Taipei 112304, Taiwan

**Keywords:** luminal type breast cancer, Asian, adjuvant! online, recurrence, gene expression profile

## Abstract

**Simple Summary:**

Multi-gene expression assays have been advocated for treatment decision in breast cancer management. The most commonly used assays such as Oncotype DX, MammaPrint, which were developed from the Western population, were especially designed for the prognostication of early stage luminal-type breast cancer. The tabulation of multi-gene expression assay and clinical risk has become the research interest recently. The 23-gene signature was purposed for the Asian population and was validated for the discriminative ability regarding 5-year relapse-free survival and the objective of this study was to evaluate the performance across distinct clinical risk groups.

**Abstract:**

Background: A 23-gene classifier has been developed based on gene expression profiles of Taiwanese luminal-like breast cancer. We aim to stratify risk of relapse and identify patients who may benefit from adjuvant chemotherapy based on genetic model among distinct clinical risk groups. Methods: There were 248 luminal (hormone receptor-positive and human epidermal growth factor receptor II-negative) breast cancer patients with 23-gene classifier results. Using the modified Adjuvant! Online definition, clinical high/low-risk groups were tabulated with the genetic model. The primary endpoint was a recurrence-free interval (RFI) at 5 years. Results: There was a significant difference between the high/low-risk groups defined by the 23-gene classifier for the 5-year prognosis of recurrence (16 recurrences in high-risk and 3 recurrences in low-risk; log-rank test: *p* < 0.0001). Among the clinically high-risk group, the 5-year RFI of high risk defined by the 23-gene classifier was significantly higher than that of the low-risk group (15 recurrences in high-risk and 2 recurrences in low-risk; log-rank test: *p* < 0.0001). Conclusion: This study showed that 23-gene classifier can be used to stratify clinically high-risk patients into distinct survival patterns based on genomic risks and displays the potentiality to guide adjuvant chemotherapy. The 23-gene classifier can provide a better estimation of breast cancer prognosis which can help physicians make a better treatment decision.

## 1. Introduction

Breast cancer is the most common female malignancy and ranks fourth among all cancer mortality in Taiwan [1]. Clinical outcomes of early breast cancers treated with curative intension have been improved enormously, mainly due to screening mammography for early detection and adjuvant systemic therapy for high-risk patients. Breast cancer is subdivided into immunohistochemistry (IHC) subtypes based on the status of estrogen receptor (ER), progesterone receptor (PR) and human epidermal growth factor receptor II (HER2). The IHC assays, in combination with anatomical stages (tumor size, regional nodal and distant organ metastasis), pathological features such as histological type and nuclear grade and an IHC-based proliferative marker, Ki67 (coded by the gene *MKI67*) not only determine which systemic therapy should be prescribed (predictive markers), but also serve as prognostic biomarkers forecasting long-term treatment outcomes [2,3].

Among all breast cancer molecular subtypes, luminal breast cancers (defined by ER and/or PR-positive and HER2-negative) enjoy the best survival rate and are the only subtype which may benefit from long-term endocrine therapy and may be spared from cytotoxic chemotherapy if residual risk following curative surgery is low enough and endocrine therapy alone can counteract the risk of recurrence [4,5].

Multi-gene expression assays (MGAs), initially adapted microarrays as the platform interrogating whole transcriptome and then commercialized with reserve transcription-polymerase chain reaction (RT-PCR), digital RNA counting (NanoString Technologies, Inc, South Lake Union, Seattle, WA), and next-generation sequencing (NGS)-based RNA sequencing have been advocated for hormone receptor (HR)-positive and HER2-negative early breast cancers for risk stratification, and serve as a decision-making tool to avoid chemotherapy. To name important ones, the Amsterdam (70-gene) and Rotterdam (76-gene) signatures, Genomic Grade Index (GGI), intrinsic subtypes (with the latest version of PAM50^®^) and Recurrence Score (21-gene) [6]. It deserves notice that both the Oncotype DX^®^ (21-gene, Exact Sciences Cooperation, Madison, WI) and MammaPrint^®^ (70-gene, Agendia Precision Oncology, NT Amsterdam, Netherlands) have been endorsed by guidelines from international societies such as the American Society of Clinical Oncology (ASCO) and the National Cancer Comprehensive Network (NCCN) with periodic focused updates as being prognostic (70-gene signature) or both prognostic and predictive (21-gene) for early stage luminal breast cancers [7,8].

Our published 23-gene signature (RecurIndex^®^, Amwise Diagnostics PTE. LTD, Taipei, Taiwan) has been proposed to classify breast cancers into high- and low-risk following curative surgery, and significantly discriminative 5-year relapse-free survival patterns were observed among 473 luminal Taiwanese breast cancers; gene expression scores with accompanied clinical variables (diagnosed age, tumor size and nodal stage) were used for risk-predictive model construction. Hazard ratios of 5.63 (95% confidence interval 2.77–11.5) and 8.02 (3.52–18.3) for high-risk subjects were reported for the genetic and clinical-genetic models, respectively [9].

Recently, with the publication of large randomized controlled trials of the MINDACT and TAILORx, the tabulation of MGA and clinical risk groups has become a major interest among clinicians and scientists engaged in breast cancer management and research [10,11,12]. The criterion of clinical risk grouping is largely based on the modified Adjuvant! Online, or the Dutch clinical risk scale [10,13]. The aim of this study is to evaluate the prognostic performance of the 23-gene signature among Taiwanese breast cancer patients with clinical high and low risk. 

## 2. Materials and Methods 

### 2.1. Study Population

**Figure 1:**
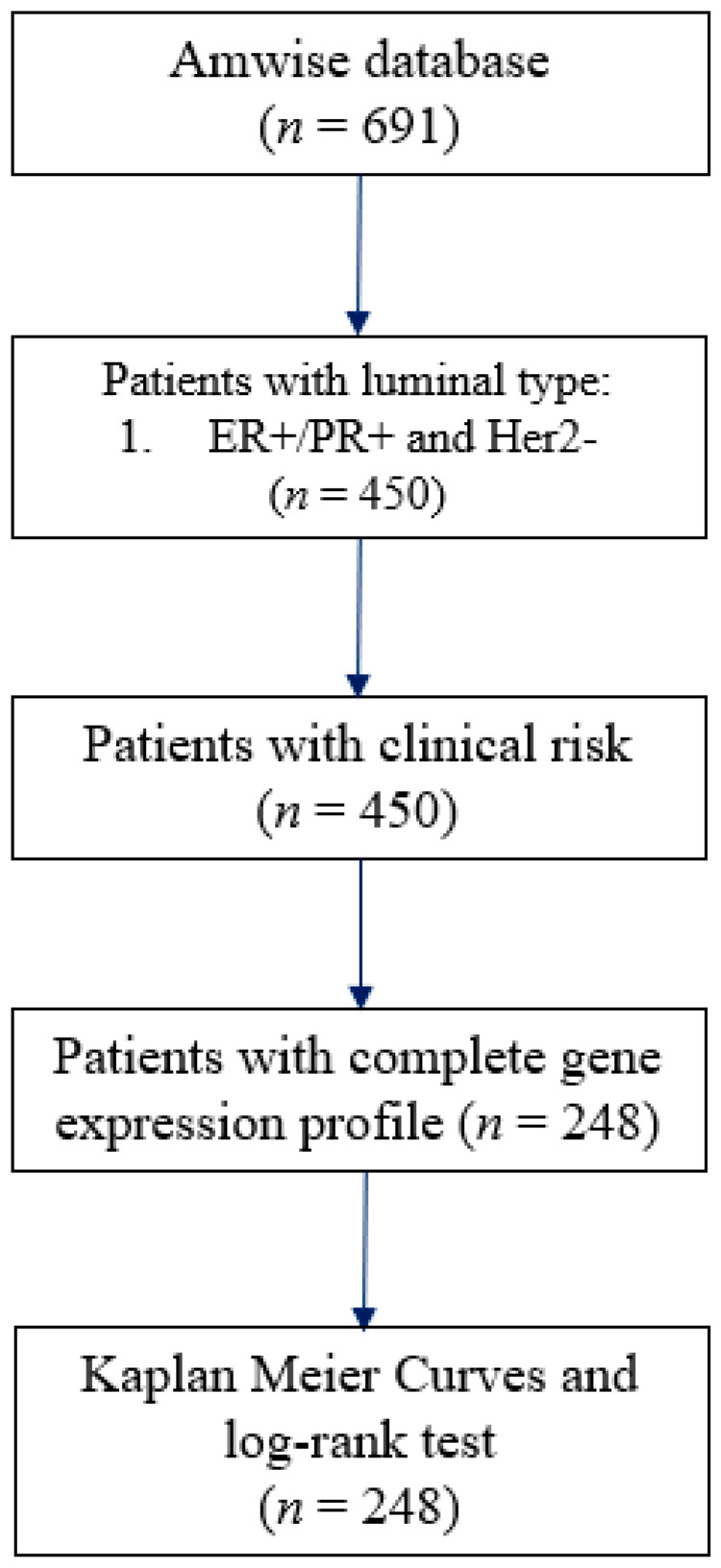
Consolidated standard of reporting trials (CONSORT) for this study to perform the workflow of this study.

The Amwise database (Amwise Diagnostics PTE. LTD., Singapore) comprised patients with breast cancer from multiple medical centers in Taiwan. All patients enrolled in the Amwise database received a standard of care including breast conserving surgery or mastectomy. The CONSORT (consolidated standard of reporting trials) diagram shows the workflow for subject selection (Figure 1). The following were the inclusion criteria: (i) luminal-like (HR positive, HER2 negative) breast cancers, (ii) complete clinical information (age, tumor grade, nodal status, and tumor size), and (iii) complete genetic data (23-gene). Exclusion criteria were: (i) patients with pre-operative chemotherapy or radiotherapy, and (ii) patients without follow-up information. In this study, there were 40% samples in the Amwise database involved in the building of the 23-gene classifier. The clinical data we collected were from the electronic medical record (EMR) from each medical center we collaborated with to obtain the treatment, follow-up, and personal information. For the extraction of gene-expression data, the reverse-transcriptase (RT) quantitative polymerase chain reaction (qPCR) technique was used to measure the gene expression of the target 23 genes by using the total RNA isolate from the formalin-fixed paraffin-embedded (FFPE) tumor tissue.

### 2.2. The 23-Gene Classifier

**Figure 2:**
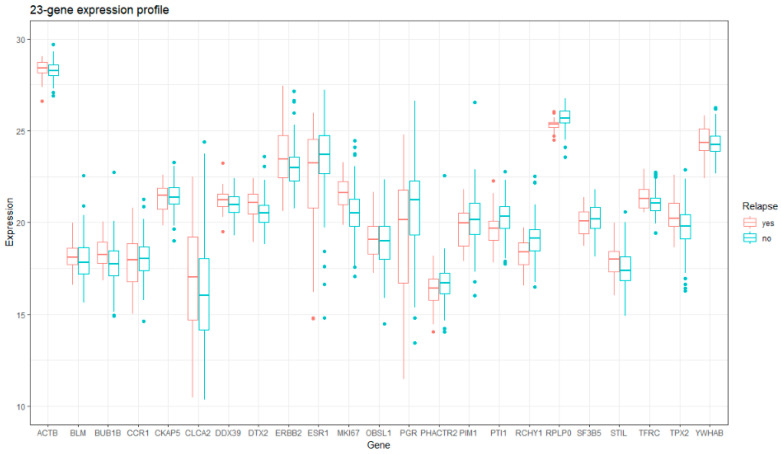
The boxplot for the visualization of 23-gene expression divided by recurrence status. Red: recurrence; blue: no recurrence; x-axis: 23 genes; y-axis: gene expression.

Development of the 23-gene classifier has been described elsewhere [9,14]. This classifier is an MGA, which interrogates functionalities associated with cell cycl and proliferation, oncogenic processes, inflammation and immune response, apoptosis and metabolism. The 23 genes panel comprised of *BLM*, *BUB1B*, *CCR1*, *CKAP5*, *CLCA2*, *DDX39*, *DTX2*, *ERBB2*, *ESR1*, *MKI67*, *OBSL1*, *PGR*, *PHACTR2*, *PIM1*, *PTI1*, *RCHY1*, *SF3B5*, *STIL*, *TPX2*, and *YWHAB* along with three housekeeping genes *ACTB*, *RPLP0*, and *TFRC.* The comparison of the gene list with other MGAs can be found in Supplementary Table (Table S1). Figure 2 summarizes the 23-gene signature. In the previous study, logistic regression with leave-one-out cross-validation (LOOCV) was performed to build a prognostic classifier [9]. First, the Ct number of 23 genes will be normalized by Equation (1) to the gene expression. Second, 23-gene signature, without housekeeping genes, was the input of the well-trained 23-gene classifier, and the output was the probability of recurrence by Equation (2). The 23-gene classifier is advocated for realizing the prognosis of recurrence for luminal breast cancers 5 years post-operatively.

(1) $$\Delta Ct=25-Ct\;\left(Gene\;of\;interest\right)+Ct\left(\frac{\left(ACTB+RPLP0+TFRC\;\right)}{3}\right)$$

(2) $$\frac{p}{1-p}=\exp\left(\beta_{0}+\beta_{1}\times Gene_{1}+\beta_{2}\times Gene_{2}+\ldots +\beta_{20}\times Gene_{20}\right)$$

where *p* is the probability of recurrence.

### 2.3. Prognostic and Statistical Analysis

The 23-gene classifier was used to determine the risk of breast cancer recurrence and provided genomic information beyond clinical risk provided by the modified Adjuvant! Online [10], which is a web tool incorporating patients’ characteristics and tumor features for estimation of relapse and survival. 

The primary endpoint of current study was a relapse-free interval (RFI) at 5 years, evaluated by Kaplan–Meier method and log-rank test between the defined high-/low-risk group. A univariate and multivariate Cox proportional hazards model was conducted to evaluate the performance of clinical and genetic model adjusted for various covariates including age group (<40, 40–60, >60), lymphovascular invasion (LVI, prominent/present versus focal/absent) and chemotherapy (with versus without). 

In Model 1 and Model 2, Cox regression was conducted for the 23-gene classifier and clinical risk groups, respectively, while Model 3 evaluated both. To investigate the incremental predictive power of the 23-gene classifier across clinical risk groups, the interaction term between clinical risk groups and the 23-gene classifier was added in Model 4. Furthermore, a subgroup analysis was conducted within each clinical risk group to evaluate the prognostic power of the 23-gene classifier among patients with and without chemotherapy. All statistical analyses were conducted with R version 4.0.2 software, with *p*-value < 0.05 as statistically significant.

## 3. Results

### 3.1. Baseline Demography of Enrolled Population

A total of 248 patients were included in this study (Table 1). The median follow-up was 67.70 (interquartile range [IQR): 43.33, 97.55) months. The majority (*n* = 168, 67.7%) was between the age of 40 and 60, 62 (25.0%) were older than 60, and 18 (7.3%) were younger than 40. A total of 190 (76.6%) was absent LVI whereas 58 (23.4%) patients presented prominent or focal LVI. Most patients were at N0 (*n* = 178, 71.8%), 59 (23.8%) at N1, and only 11 (4.4%) at N2 nodal stage. Regarding tumor stage, most patients were at T1 (*n* = 114, 45.9%) or T2 (*n* = 123, 49.6%), and only 11 (4.4%) at T3. In addition, 95 patients had tumor at grade I (38.3%), 130 at grade II (52.4%) and 23 at grade III (9.3%). Almost all the subjects (240 or 96.77%) received hormonal therapy; only 3.23% of participants did not receive hormonal therapy. There were 156 (62.9%) patients without adjuvant chemotherapy, whereas 92 (37.1%) patients receiving adjuvant chemotherapy. A total of 179 (72.2%) patients were with clinical high risk, of which 23 (12.9%) relapsed within 5 years; 69 (27.8%) were of the clinical low-risk group, of which 4 (5.8%) relapsed within 5 years, based on the modified Adjuvant! Online criteria. Clinical characteristics of enrolled patients for evaluating the treatment status are detailed in Table 2 and Table 3. These tables showed that factors such as tumor stage (*p* < 0.001), nodal stage (*p* < 0.001) and tumor grade (*p* = 0.008) of patients with chemotherapy were worse than of those without. Regarding radiotherapy, only tumor stage (*p* = 0.024) and follow-up time (*p* < 0.001) were significantly different between those with and without radiotherapy.

### 3.2. The Clinical Performance between Two Prognosis Tools

Table 4 showed a good partition from 23-gene classifier in the prediction of relapse. Either accuracy or NPV was over than 85% (accuracy, 0.855 and NPV, 0.815). The performance of Modified Adjuvant! Online (Table 5) showed poor partition in this population (accuracy, 0.355 and NPV, 0.294). Regarding the F1 score, the metric of the 23-gene classifier was 0.913, which was much higher than the value of Modified Adjuvant! Online (F1-score, 0.448).

### 3.3. RFI Analysis and Cox Proportional Hazards Regression Model

**Figure 3:**
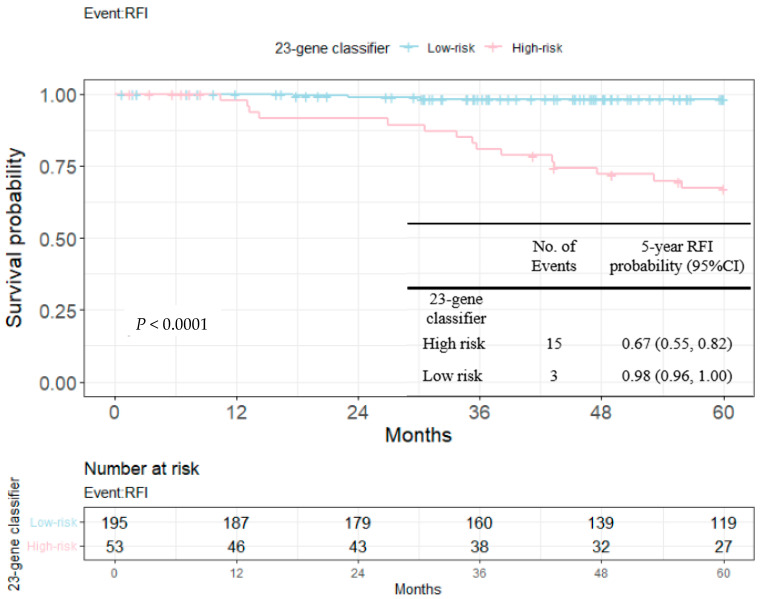
Kaplan–Meier plot for RFI within 5 years to compare the partition of 23-gene classifier. Blue: genetic low-risk group; red: genetic high-risk group.

**Figure 4:**
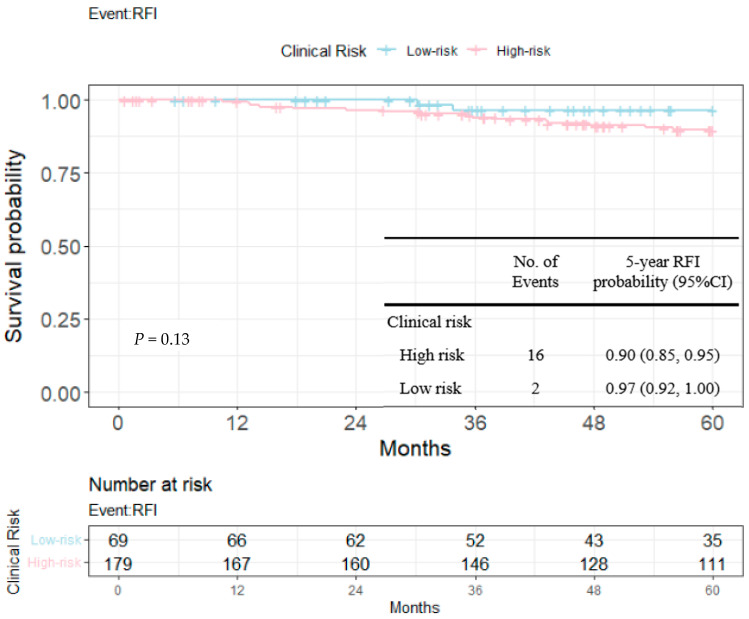
Kaplan–Meier plot for RFI within 5 years to compare the partition of clinical risk. Blue: clinically low-risk group; red: clinically high-risk group.

**Figure 5:**
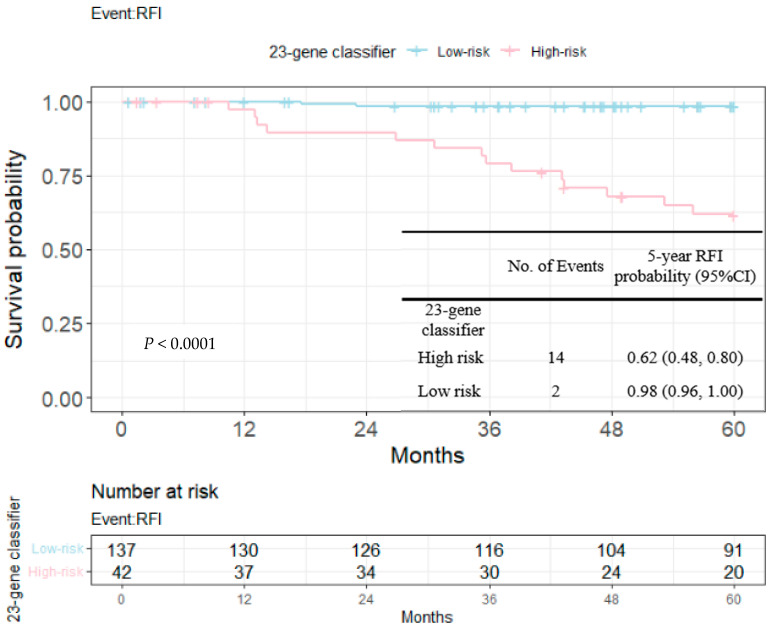
Kaplan–Meier plot for RFI within 5 years in clinically high-risk group to compare the partition of 23-gene classifier. Blue: genetic low-risk group; red: genetic high-risk group.

**Figure 6:**
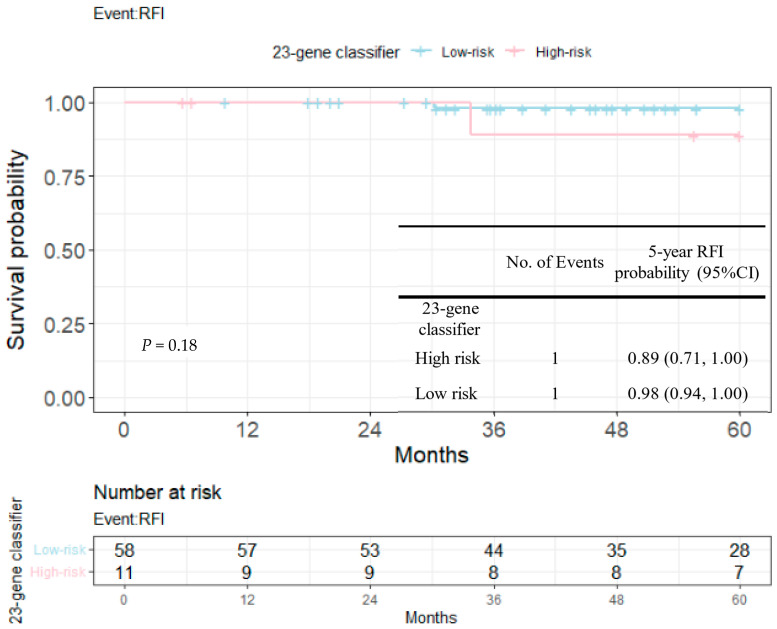
Kaplan–Meier plot for RFI within 5 years in clinically low-risk group to compare the partition of 23-gene classifier. Blue: genetic low-risk group; red: genetic high-risk group.

**Figure 7:**
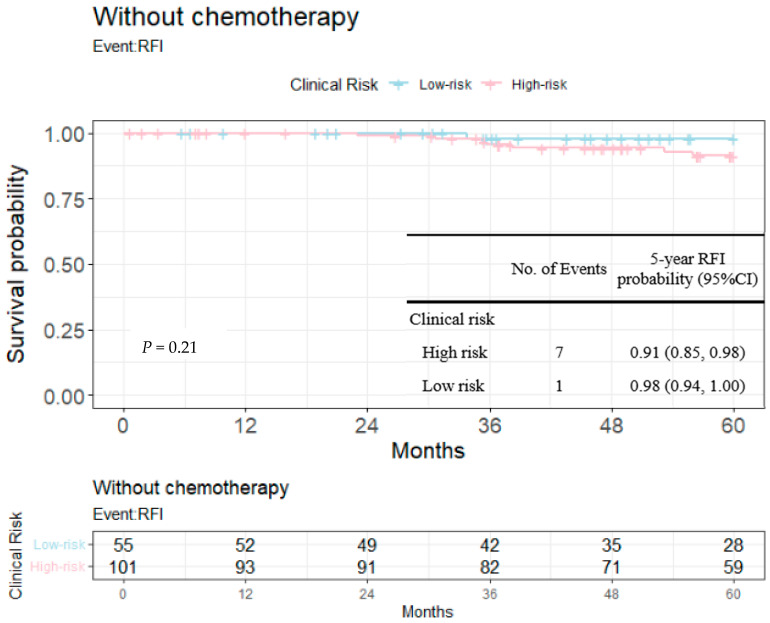
Kaplan–Meier plot for the RFI among patients without chemotherapy within 5 years to compare the partition of clinical risk. Blue: clinically low-risk group; red: clinically high-risk group.

**Figure 8:**
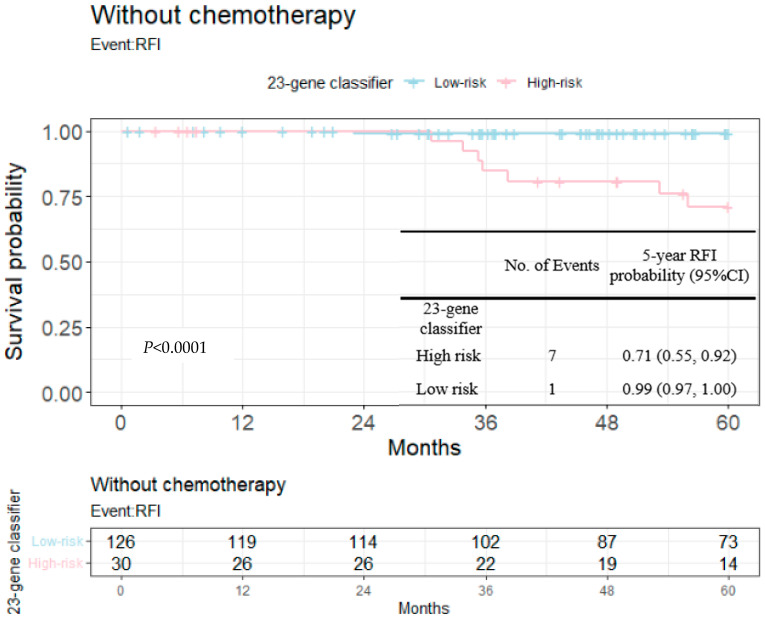
Kaplan–Meier plot for the RFI among patients without chemotherapy within 5 years to compare the partition of 23-gene classifier. Blue: genetic low-risk group; red: genetic high-risk group.

RFIs stratified by the 23-gene classifier and clinical risk groups are shown in Figure 3 and Figure 4. The recurrence-free probability between the high-/low-risk group defined by the genetic classifier was significant (*p* < 0.0001), and was 0.67 (95% CI: 0.55, 0.82) for high- and 0.98 (95% CI: 0.96, 1.00) for low-risk group, respectively. On the other hand, the 5-year recurrence-free probability was 0.90 (95% CI: 0.85, 0.95) and 0.97 (95% CI: 0.92, 1.00) for the clinical high- and low-risk group. Among clinically high-risk patients, the recurrence-free probability of the genetic high-risk group was 0.62 (95% CI: 0.48, 0.80) and was 0.98 for the genetic low-risk group (95% CI: 0.96, 1.00) (Figure 5). Figure 6 shows the 5-year RFI in the clinically low-risk group. Among patients without chemotherapy (Figure 7 and Figure 8), the recurrence-free probability of clinically high-risk group was 0.91 (95% CI: 0.85, 0.98), which was worse than that of clinically low-risk group, but was not statistically significant (0.98 (95% CI: 0.94, 1.00), log-rank test: 0.21). On the other hand, the 5-year recurrence-free probability of the genetic high-risk group was 0.71 (95% CI: 0.55, 0.92) at 5-years and was 0.99 (95% CI: 0.97, 1.00) for the genetic low-risk group without adjuvant chemotherapy.

Table 6 summarizes the results of Cox proportional hazards regression for the 23-gene classifier and clinical risk groups. In the univariate analysis, only the 23-gene classifier was found to have a significant effect on recurrence within 5 years (hazard ratio: 20.9 [95% CI: 6.04, 72.1]) and the effect of clinically high-risk group was borderline (hazard ratio: 2.92 [95% CI: 0.67, 12.7]). In Model 1, after controlling potential confounders, the 23-gene classifier remained a significant predictor for recurrence status within 5 years (hazard ratio: 10.5 [95%CI: 2.65, 41.8]). The clinical risk groups were also an independent prognostic factor in Model 2 (hazard ratio: 1.59 [95% CI: 0.30, 8.35]). In Model 3, a multivariate Cox regression model comprised both the 23-gene classifier and clinical risk groups, and the 23-gene classifier remained an independent predictor for the 5-year recurrence (hazard ratio: 10.5 [95% CI: 2.63, 42.2]). In Model 4, the interaction term tabulating clinical risk groups and the 23-gene classifier was not statistically significant (*p* = 0.6).

## 4. Discussion

In the past two decades, gene expression profiling has re-defined breast cancer as a molecularly heterogeneous disease entity which displays a broad spectrum of alternations in transcriptome, and sub-classifications that not only enhance molecular taxonomy, but have provided prognostic information pertaining to survival after curative therapy [15]. In addition to the well-known published MGAs, the 23-gene signature has been validated for its discriminating ability in addition to pathological prognostic factors such as tumor size and nodal status [9]. In one study, the prognostic discrepancy in 5-year relapse-free survival was evidenced between the predicted high- and low-risk groups [9,14]. Chronologically and collectively, the proposed signature has been validated across microarray and RT-PCR platforms [9,14].

It is not a coincidence that several MGAs have been used in combination with clinical risk factors such as tumor size and nodal status. The EPclin score composes a 12-gene molecular score and clinical features, while the PAM50-based risk of recurrence (ROR) score adopts a 50-gene signature as well as clinical features [16,17]. These second-generation MGAs are capable of predicting 10-year distant recurrence [18,19]. On the other hand, for the two signatures with purely genetic scores, the latest clinical trials all incorporated clinical risk groups stratifying targeted populations; both the MINDACT (clinically high- and genomic low-risk group) and RxPONDER identified a subset of post-menopausal (age > 50-year-old and recurrence score < 25) pN1 patients who may be safely spared from cytotoxic chemotherapy [10,20]. For pre-menopausal (age < 50-year-old) patients, the situation is much more complicated, as there is still a substantial benefit to chemotherapy (~5%) for the clinically high and genomic low MINDACT population, as well as those of recurrence score 16–20 with clinically high risk, and all risk groups with recurrence score > 21 from TAILORx trial [6,10,11]. The Dutch clinical risk criteria (low-risk definition: age > 35 years and [grade 1 with tumor ≤3cm, grade 2 with tumor ≤2cm, or grade 3 with tumor ≤1cm]) and the modified Adjuvant! Online criteria have been used in clinical risk stratification for both the MammaPrint and Oncotype DX^®^ [21,22].

In the current study, we evaluated the prognostic value of the 23-gene signature from an unselected Taiwanese early breast cancer cohort across distinct clinical risk groups. Both genetic and clinical risk groups were prognostic as shown in Figure 3, 4 and Table 4, while the slightly smaller *p*-value indicated the better discriminative ability of the purposed genetic score than clinical risk groups (univariate Cox’s model and multi-variate model 1 and 2). Figure 5 and Figure 6 shows that among clinically high-risk group patients (*n* = 179), the 23-gene signature remained prognostic, which did not hold true for clinically low-risk counterparts (*n* = 69). Among the clinically high-risk sub-population, there were 14 events out of 42 genetically high-risk subjects defined by the 23-gene classifier, while only 2 events were observed during the 5-year follow up period from 137 genetically low-risk subjects predicted by the signature, resulting in a highly significant *p*-value of 0.0001 (Figure 5). Prognostic discrimination of the 23-gene classifier diminished for patients with a low clinical risk, indicating that these subjects might not be the targeted population of the purposed signature. 

We further dug into the impact of risk prediction upon 5-year recurrence-free intervals among patients not receiving adjuvant chemotherapy (*n* = 156). As noted in Figure 7 and Figure 8, worse survival was observed for those predicted as high risk by clinical risk groups or the 23-gene classifier, but without chemotherapy. Among all patients not receiving chemotherapy (Table 4 shows that patients with a larger tumor, advanced pN stage and a higher nuclear grade tended to receive chemotherapy while the presence of LVI was only borderline statistically significant), prognostic discrimination was more pronounced for the 23-gene defined risk groups (7 out of 30 genetically high-risk patients experienced events, while only 7 out of 101 clinically high-risk patients had events). In other words, more patients were categorized into high-risk group by genetic model rather than the clinical model among patients without chemotherapy. It deserves notice that there were still more patients experiencing relapse even after chemotherapy, indicating an unmet need of increased risk not covered by adjuvant systemic therapy; more patients (30% versus 20%) in the genetically high-risk group received chemotherapy, but did not reach a statistical significance (Table 4).

In summary, both the genetic and clinical risk groups were prognostic in terms of 5-year recurrence-free interval, while the 23-gene signature was more prognostic than the clinical risk groups. Among clinically high-risk patients, the prognostic power of the 23-gene signature remains, further indicating that these patients were the targeted population for the use of MGA. Among patients not receiving adjuvant therapy, both the 23-gene classifier and clinical risk groups were prognostic, while the genetic risk group was more predictive for 5-year recurrence events. 

There were some limitations to the current study. First, the observational rather than interventional design limited the predictive power of adjuvant chemotherapy benefits from the purposed signature, although the 23-gene classifier was prognostic among patients not receiving chemotherapy. The allocation of chemotherapy in the current study was determined by clinicians, which might be correlated with factors defining clinical risk groups, such as tumor size and nodal status. Second, the retrospective study design also hampered the evidence level of the deduced conclusion. Third, the modest sample size further limited subgroup analyses regarding each tabulation of genetic and clinical risk groups, due to the paucity of cases in each stratum. 

## 5. Conclusions

In conclusion, this study ascertained that the 23-gene classifier could stratify early breast cancer patients with clinical high risk into distinct survival patterns, and have the potentiality to support decision making in adjuvant chemotherapy. This MGA can provide a better estimation of breast cancer prognosis which can help physicians with precise management of luminal breast cancers.

## 6. Patents

Amwise holds the patent related to the content of this manuscript (Taiwan patent application number: 109132402; China patent application number: 202011103766.4).

## Figures and Tables

**Table 1:** Basic clinical characteristics of enrolled luminal-like breast cancer patients.

Characteristic	Overall	Clinical Risk		
	N = 248 *^1^*	Low, N = 69 *^1^*	High, N = 179 *^1^*	*p*-Value *^2^*
**Age**				0.8
40–60	168 (67.74%)	49 (71.01%)	119 (66.48%)	
>60	62 (25.00%)	16 (23.19%)	46 (25.70%)	
<40	18 (7.26%)	4 (5.80%)	14 (7.82%)	
**Tumor stage**				<0.001
T1	114 (45.97%)	63 (91.30%)	51 (28.49%)	
T2	123 (49.60%)	6 (8.70%)	117 (65.36%)	
T3	11 (4.44%)	0 (0.00%)	11 (6.15%)	
**N stage**				<0.001
N0	178 (71.77%)	65 (94.20%)	113 (63.13%)	
N1	59 (23.79%)	4 (5.80%)	55 (30.73%)	
N2	11 (4.44%)	0 (0.00%)	11 (6.15%)	
**LVI**				<0.001
No	190 (76.61%)	66 (95.65%)	124 (69.27%)	
Yes	58 (23.39%)	3 (4.35%)	55 (30.73%)	
**Grade**				<0.001
I	95 (38.31%)	21 (30.43%)	74 (41.34%)	
II	130 (52.42%)	48 (69.57%)	82 (45.81%)	
III	23 (9.27%)	0 (0.00%)	23 (12.85%)	
**Chemotherapy**				<0.001
No	156 (62.90%)	55 (79.71%)	101 (56.42%)	
Yes	92 (37.10%)	14 (20.29%)	78 (43.58%)	
**Radiotherapy**				0.10
No	145 (58.47%)	46 (66.67%)	99 (55.31%)	
Yes	103 (41.53%)	23 (33.33%)	80 (44.69%)	
**Hormonal therapy**				0.11
No	8 (3.23%)	0 (0.00%)	8 (4.47%)	
Yes	240 (96.77%)	69 (100.0%)	171 (95.53%)	
**Relapse**				0.11
No	221 (89.11%)	65 (94.20%)	156 (87.15%)	
Yes	27 (10.89%)	4 (5.80%)	23 (12.85%)	
**23-gene classifier**				0.2
Low	195 (78.63%)	58 (84.06%)	137 (76.54%)	
High	53 (21.37%)	11 (15.94%)	42 (23.46%)	
**Follow-up**	67.70 [43.33, 97.55]	62.23 [36.26, 102.77]	68.46 [45.31, 92.70]	0.8

*^1^* n (%); median [*25*%, *75*%] ^2^ Pearson’s Chi-squared test; Fisher’s exact test; Wilcoxon rank sum test.

**Table 2:** Basic clinical characteristics among patients with/without chemotherapy.

	Chemotherapy		
Characteristic	No, N = 156 *^1^*	Yes, N = 92 *^1^*	*p*-Value *^2^*
**Age**			0.3
40–60	101 (64.74%)	67 (72.83%)	
>60	44 (28.21%)	18 (19.57%)	
<40	11 (7.05%)	7 (7.61%)	
**Tumor stage**			<0.001
T1	83 (53.21%)	31 (33.70%)	
T2	71 (45.51%)	52 (56.52%)	
T3	2 (1.28%)	9 (9.78%)	
**N stage**			<0.001
N0	129 (82.69%)	49 (53.26%)	
N1	25 (16.03%)	34 (36.96%)	
N2	2 (1.28%)	9 (9.78%)	
**LVI**			0.044
No	126 (80.77%)	64 (69.57%)	
Yes	30 (19.23%)	28 (30.43%)	
**Grade**			0.008
I	66 (42.31%)	29 (31.52%)	
II	82 (52.56%)	48 (52.17%)	
III	8 (5.13%)	15 (16.30%)	
**Relapse**			0.012
No	145 (92.95%)	76 (82.61%)	
Yes	11 (7.05%)	16 (17.39%)	
**Clinical risk**			<0.001
Low	55 (35.26%)	14 (15.22%)	
High	101 (64.74%)	78 (84.78%)	
**23-gene classifier**			0.3
Low	126 (80.77%)	69 (75.00%)	
High	30 (19.23%)	23 (25.00%)	
**Follow-up**	67.03 [40.64, 99.79]	68.43 [44.78, 89.02]	0.8

*^1^ n* (%); median [*25*%, 75%] *^2^* Pearson’s Chi-squared test; Fisher’s exact test; Wilcoxon rank sum test.

**Table 3:** Basic clinical characteristics among patients with/without radiotherapy.

	Radiotherapy		
Characteristic	No, N = 145 *^1^*	Yes, N = 103 *^1^*	*p*-Value *^2^*
**Age**			0.076
40–60	96 (66.21%)	72 (69.90%)	
>60	42 (28.97%)	20 (19.42%)	
<40	7 (4.83%)	11 (10.68%)	
**Tumor stage**			0.024
T1	68 (46.90%)	46 (44.66%)	
T2	75 (51.72%)	48 (46.60%)	
T3	2 (1.38%)	9 (8.74%)	
**N stage**			0.6
N0	104 (71.72%)	74 (71.84%)	
N1	36 (24.83%)	23 (22.33%)	
N2	5 (3.45%)	6 (5.83%)	
**LVI**			>0.9
No	111 (76.55%)	79 (76.70%)	
Yes	34 (23.45%)	24 (23.30%)	
**Grade**			0.2
I	51 (35.17%)	44 (42.72%)	
II	83 (57.24%)	47 (45.63%)	
III	11 (7.59%)	12 (11.65%)	
**Relapse**			0.2
No	132 (91.03%)	89 (86.41%)	
Yes	13 (8.97%)	14 (13.59%)	
**Clinical risk**			0.10
Low	46 (31.72%)	23 (22.33%)	
High	99 (68.28%)	80 (77.67%)	
**23-gene classifier**			0.12
Low	109 (75.17%)	86 (83.50%)	
High	36 (24.83%)	17 (16.50%)	
**Follow-up**	77.01 [48.20, 102.77]	56.75 [30.90, 76.11]	<0.001

*^1^ n* (%); median [25%, 75%] *^2^* Pearson’s Chi-squared test; Fisher’s exact test; Wilcoxon rank sum test.

**Table 4:** Confusion matrix with clinical performance metrics for 23-gene classifier.

Characteristic	Clinical Outcome: Relapse		Total
	Yes	No	
**23-gene classifier** **(high/low)**			
High	22	5	27
Low	31	190	221
**Total**	53	195	248

Accuracy, 0.855; sensitivity, 0.415; specificity, 0.974; PPV, 0.815; NPV, 0.859; F1 score: 0.913.

**Table 5:** Confusion matrix with clinical performance metrics for Modified Adjuvant! Online.

Characteristic	Clinical Outcome: Relapse		Total
	Yes	No	
**Clinical risk** **(high/low)**			
High	23	4	27
Low	156	65	221
**Total**	179	69	248

Accuracy, 0.355; sensitivity, 0.129; specificity, 0.942; PPV, 0.852; NPV, 0.294; F1 score, 0.448.

**Table 6:** Cox proportional hazards regression for RFI within 5 years.

Characteristic	Univariate Analysis			Model 1			Model 2			Model 3			Model 4		
	HR *^1^*	95% CI *^1^*	*p*-Value	HR *^1^*	95% CI *^1^*	*p*-Value	HR *^1^*	95% CI *^1^*	*p*-Value	HR *^1^*	95% CI *^1^*	*p*-Value	HR *^1^*	95% CI *^1^*	*p*-Value
Age															
40–60	—	—		—	—		—	—		—	—		—	—	
>60	1.31	0.45, 3.82	0.625	1.46	0.49, 4.36	0.5	1.25	0.42, 3.69	0.7	1.45	0.48, 4.33	0.5	1.47	0.49, 4.42	0.5
<40	2.96	0.81, 10.7	0.100	2.50	0.59, 10.5	0.2	3.31	0.84, 13.1	0.087	2.39	0.56, 10.2	0.2	2.31	0.54, 9.86	0.3
LVI															
No	—	—		—	—		—	—		—	—		—	—	
Yes	0.69	0.20, 2.38	0.555	0.37	0.09, 1.56	0.2	0.17	0.04, 0.68	0.012	0.37	0.09, 1.55	0.2	0.38	0.09, 1.62	0.2
N stage															
N0	—	—		—	—		—	—		—	—		—	—	
N1	4.08	1.37, 12.1	0.012	3.24	0.67, 15.6	0.14	6.55	1.62, 26.5	0.008	2.89	0.54, 15.5	0.2	2.73	0.51, 14.5	0.2
N2	15.8	4.82, 51.8	<0.001	7.65	1.40, 41.8	0.019	26.6	5.65, 126	<0.001	6.77	1.12, 40.9	0.037	6.25	1.03, 38.0	0.047
Tumor grade															
Grade I	—	—		—	—		—	—		—	—		—	—	
Grade II	3.54	1.00, 12.5	0.050	1.40	0.35, 5.55	0.6	2.40	0.65, 8.92	0.2	1.43	0.36, 5.77	0.6	1.38	0.34, 5.58	0.6
Grade III	5.18	1.04, 25.7	0.044	2.00	0.34, 11.8	0.4	5.10	0.89, 29.0	0.067	1.91	0.32, 11.3	0.5	1.78	0.30, 10.7	0.5
Chemotherapy															
No	—	—		—	—		—	—		—	—		—	—	
Yes	2.08	0.82, 5.27	0.122	0.69	0.19, 2.52	0.6	0.63	0.20, 1.98	0.4	0.69	0.19, 2.48	0.6	0.69	0.19, 2.46	0.6
23-gene classifier															
Low	—	—		—	—					—	—		—	—	
High	20.9	6.04, 72.1	<0.001	10.5	2.65, 41.8	<0.001				10.5	2.63, 42.2	<0.001	5.60	0.35, 90.8	0.2
Clinical risk															
Low	—	—					—	—		—	—		—	—	
High	2.92	0.67, 12.7	0.153				1.59	0.30, 8.35	0.6	1.38	0.25, 7.80	0.7	0.85	0.07, 9.92	0.9
Interaction of clinical risk and 23-gene classifier															
High * High													2.33	0.09, 62.2	0.6

*^1^* HR = hazard ratio, CI = confidence interval, * = interaction term between genetic high-risk and clinical high-risk; Model 1: multiple Cox proportional model including age, LVI, nodal stage, tumor grade, chemotherapy, and 23-gene classifier. Model 2: multiple Cox proportional model including age, LVI, nodal stage, tumor grade, chemotherapy and clinical risk. Model 3: multiple Cox proportional model including age, LVI, nodal stage, tumor grade, chemotherapy, 23-gene classifier and clinical risk. Model 4: multiple Cox proportional model including age, LVI, nodal stage, tumor grade, chemotherapy, 23-gene classifier, clinical risk and interaction of 23-gene classifier and clinical risk.

## Data Availability

The original contributions presented in the study are included in the article/supplementary material, further inquiries can be directed to the corresponding author/s.

## Supplementary Materials

The following supporting information can be downloaded at: https://www.mdpi.com/article/10.3390/cancers14246263/s1, Table S1: Comparison of the gene list with other MGA.

## Abbreviations

ER	estrogen receptor
FFPE	formalin-fixed paraffin-embedded
HER2	human epidermal growth factor receptor 2
HR	hormone receptor
IHC	immunohistochemistry
LVI	lymphovascular invasion
MGA	multi-gene expression assays
PR	progesterone receptor
RFI	recurrence-free interval
ROC	receiver operating characteristic
RT-PCR	reverse transcriptase-polymerase chain reaction
