# Supplementary material for: Comparing Genetic Risk and Clinical Risk Classification in Luminal-like Breast Cancer Patients Using a 23-Gene Classifier
Source: Cancers (Basel). 2022 Dec 19;14(24):6263. doi: 10.3390/cancers14246263 (PMC9776657; doi:10.3390/cancers14246263)
Supplement: Supplementary file 1 [file cancers-14-06263-s001.zip › cancers-1980446-supplementary.pdf]

Table S1. Comparison of the gene list with other MGA

MammaPrint 70 genes	PAM 50 50 genes	Oncotype Dx 21 genes	Endopredict 11 genes	23-gene classifier
ACADS	ACTR3B	ACTB	AZGP1	DDX39
AKAP2	ANLN	BAG1	BIRC5	BUB1B
ALDH4	BAG1	BCL2	CALM2	CCR1
AP2B1	BCL2	BIRC5	DHCR7	STIL
ASNS	BIRC5	CATL2	IL6ST	BLM
BBC3	BLVRA	CD68	MGP	PIM1
BTG2	CCNB1	CCNB1	OAZ1	TPX2
BUB1	CCNE1	ESR1	RBBP8	PTI1
CA9	CDC20	GAPDH	RPL37A	DTX2
CCNB2	CDC6	GRB7	STC2	RCHY1
CCNE2	CDCA1	GSTM1	UBE2C	OBSL1
CEGP1	CDH3	GUS		CLCA2
CENPA	CENPF	ERBB2		CKAP5
CFFM4	CEP55	MKI67		YWHAB
COL4A2	CXXC5	MYBL2		ESR1
CP	EGFR	PGR		PGR
DC13	ERBB2	RPLPO		ERBB2
DCK	ESR1	SCUBE2		MKI67
DKFZP564D0462	EXO1	AURKA		ACTB
ECT2	FGFR4	MMP11		RPLP0
ESM1	FOXA1	TFRC		TFRC
EXT1	FOXC1			PHACTR2
FGF18	GPR160			SF3B5
FLJ10901	GRB7			
FLJ11190	KIF2C			
FLJ11354	KNTC2			
FLJ12443	KRT14			
FLJ20354	KRT17			
FLJ22477	KRT5			
FLT1	MAPT			
GCN1L1	MDM2			
GMPS	MELK			
GNAZ	MIA			
GSTM3	MKI67			
HEC	MLPH			
HSA250839	MMP11			
IGFBP5	MYBL2			
KIAA0175	MYC			
KIAA1104	NAT1			

KIAA1181	ORC6L
KIAA1442	PGR
L2DTL	PHGDH
LOC51203	PTTG1
LOC57110	RRM2
MCCC1	SFRP1
MCM6	SLC39A6
MMP9	TMEM458
MMSDH	TYMS
MP1	UBE2C
NMU	UBE2T
ORC6L	
OXCT	
PECI	
PEX12	
PGK1	
PK428	
PRC1	
QDPR	
RAB6B	
RAD21	
RFC4	
SERF1A	
SLC2A3	
SM-20	
AURKA	
STK3	
TGFB3	
TMEFF1	
UCH37	
WISP1	

	Overlapping Gene
	House keeping Gene
